# Measuring adjustment of siblings of children with disabilities: psychometric properties across translations, age groups and informants

**DOI:** 10.1080/20473869.2024.2411511

**Published:** 2024-10-09

**Authors:** Linda K. M. Veerman, Stian Orm, Krister W. Fjermestad, Torun M. Vatne, Yngvild B. Haukeland, Paula S. Sterkenburg, Agnes M. Willemen

**Affiliations:** a Clinical Child & Family Studies, Amsterdam Public Health, Vrije Universiteit Amsterdam, Amsterdam, the Netherlands; b Department of Psychology, Inland Norway University of Applied Sciences, Lillehammer, Norway; c Department of Psychology, University of Oslo, Oslo, Norway; d Frambu Resource Center for Rare Disorders, Siggerud, Norway; e The Blue Cross, Oslo, Norway; f Bartiméus, Doorn, the Netherlands

**Keywords:** Siblings, negative adjustment, questionnaire, psychometrics, invariance

## Abstract

**Objectives:**

The Negative Adjustment Scale (NAS) is used to measure adjustment to having a sibling with a disability. However, several adaptations to the scale have been made, and implementation varies across studies and countries. This study examined the psychometric properties across different versions and provides directions for future use and development of the NAS.

**Methods:**

The sample comprised 400 siblings aged 6–16 years old from Norway, the Netherlands and Belgium. Measurement invariance was assessed across age groups (8–11 vs. 12–16 years) and translations (Norwegian vs. Dutch), using a multi-group confirmatory factor analysis. The psychometric properties of the parent version were assessed among 102 parents from the Dutch sample.

**Results:**

The internal consistency of the NAS was acceptable to good in all versions. A single factor model held across age groups and translations, although higher order invariance was not supported. The child and parent report versions of the NAS did not significantly correlate.

**Conclusions:**

The NAS generally has sufficient psychometric properties. However, these vary across age groups, translations, and informants, causing the NAS to be less appropriate for comparisons between groups. Further adaptation of the NAS or developing a new scale for siblings of children with disabilities, is advised.

## Introduction

### Background

If a child has a disability, such as intellectual disabilities, chronic physical conditions, or neurodevelopmental disorders, this impacts all family members, including siblings. The impact on siblings from growing up with a child who has a disability, has been studied for decades. Both positive and negative impacts have been reported (Blamires et al. 2024; Pinquart 2023; Wofford and Carlson 2017). Siblings of children with disabilities can experience social and emotional difficulties (Hanvey, Malovic, and Ntontis 2022; Wolff et al. 2022). They display higher levels of depression symptoms and atypical development compared to siblings of children who do not have a disability (Martinez et al. 2022; Wolff, Franco, et al. 2023). At the same time, siblings of children with, for example, chronic physical conditions or autism show more prosocial behaviour than siblings of children who do not have a disability, and are described as being mature, compassionate, empathic, and resilient (Leedham, Thompson, and Freeth 2020; Pinquart 2023).

Researchers have concluded that sibling outcomes are not mainly impacted by having a sibling with a disability itself, but rather by a wide range of additional interrelated factors with bidirectional influences on the individual, family, and societal level (Kovshoff et al. 2017). The multiple factors include, for example, symptom severity and behavioural problems of the child with a disability, individual coping strategies, perceived and real social support, parental stress, and societal attitudes to disability (Marquis, Hayes, and McGrail 2019; Wolff et al. 2022). Sibling outcomes can thus only be understood in a wider context, as is further explained in the Siblings Embedded Systems Framework (Kovshoff et al. 2017).

Apart from considering the wider context associated with sibling outcomes, it is important that these outcomes are measured in an adequate, reliable, valid, and consistent way. However, psychometrically sound outcome measures for siblings do not represent the state-of-the-art, and many different outcome measures have been used across intervention studies (Wolff, Magiati, et al. 2023). A common sibling outcome includes adjustment, which has been conceptualized differently across studies (Lummer-Aikey and Goldstein 2021). Adjustment is often used in a general sense to describe psychological outcomes, containing behavioral, emotional, and social functioning (Meadan, Stoner, and Angell 2010). This can include positive aspects, such as prosocial behavior, and negative aspects, such as symptoms of psychological disorders and behavioral problems. Other sibling studies (e.g. Haukeland et al. 2015) focused on adjustment described as a process: how one appraises and adapts to an event or situation, such as having a disability, or living with a family member with a disability. In this sense, adjustment does not describe psychological outcomes, but contributes to these (Moss-Morris 2013). Sibling-tailored instruments are needed to measure what is specifically important regarding siblings’ adjustment to living with a child with a disability, and to capture its multiple aspects, in addition to more general measures, such as the Strengths and Difficulties Questionnaire (SDQ; Goodman 1997) (Hartling et al. 2014; Meadan, Stoner, and Angell 2010).

The most widely used sibling-tailored measure is the Sibling Perception Questionnaire (SPQ). This scale was originally developed to measure the affective evaluations of siblings regarding the impact living with a child with cancer has on their self-perceptions and familial and non-familial relationships (Sahler and Carpenter 1989). Researchers also used the scale for siblings of children with other disabilities, such as chronic illnesses, developmental disabilities, physical disabilities, hearing impairment, and autism (Gutierrez, Horan, and Limbers 2023). The original SPQ has four subscales: Interpersonal (how the disability affects the sibling in relation and interaction with others), Intrapersonal (how the disability personally affects the sibling, e.g. their thoughts and emotions), Communication (to what extend the sibling can talk to others), and Fear of Disease (the sibling’s fear of (catching) the disease). Because the internal consistency of the SPQ was insufficient, in a subsequent study, a revised version, the combined Negative Adjustment Scale (NAS), was developed (Lobato and Kao 2002). The NAS measures adjustment as a process, focusing on various aspects of siblings’ intra- and interpersonal adjustment to living with a child with a disability. The scale has been used with children aged 6–18 years. Often, but not in all studies, it has been completed with support from a research assistant (Gutierrez, Horan, and Limbers 2023). A parent proxy report version was created (Lobato and Kao 2002) and used for siblings aged 4 years and older (Gutierrez, Horan, and Limbers 2023). This version uses rephrased items of the original scale (e.g. ‘I wish I knew someone who understood how I am feeling’ was rephrased into ‘My child wishes he/she knew someone who understood how he/she is feeling)’.

Studies have shown promising evidence for the psychometric properties of the child version of the NAS, with higher internal consistency compared to the total scale and subscales of the original SPQ version (Gutierrez, Horan, and Limbers 2023). Furthermore, a recent study conducted by Orm et al. (2021) supported the proposed single-factor structure, and reported convergent validity with positive associations with externalizing and internalizing difficulties. A few intervention studies also showed the NAS’ sensitivity to change (Haukeland et al. 2020; Lobato and Kao 2002). Finally, Fredriksen et al. (2021) showed that sibling adjustment measured with the NAS was associated with siblings’ emotional and behavioural adjustment, indicating the relevance of the measure.

However, a review of the scale underlined that further research with more sophisticated methods and larger sample sizes is needed (Gutierrez, Horan, and Limbers 2023). Moreover, differences and unclarity exist in the implementation of the NAS, including adaptations made to Likert scales, omitted items, differences in sample characteristics (e.g. age, diagnosis), and translation procedures. For future use of the scale, it is essential to assess if these differences cause variance between groups and versions, or that the scale can validly be used across these groups and versions. The latter would allow researchers to use more complex research methods in larger, combined samples of studies that use the NAS.

### The current study

The aim of the current study was to extend the evidence of the psychometric properties of the NAS and advise the sibling field about the further use and development of this scale. First, we replicated internal consistency and factor structure analysis of the child report NAS in a larger sample. We expected to find similar results to previous studies, with acceptable internal consistency and fit for the single-factor model (Orm et al. 2021).

Second, we assessed the invariance of the scale across age groups. Invariance means that the scale measures the same construct across groups, and meaningful comparisons between groups and versions can be made (Leitgöb et al. 2023). We selected two age groups, within the range of our sample, that are in line with child development stages (Eccles 1999), namely 8–11 and 12–16 years. Demographic variables, including age, may also impact the experiences and interpretations of siblings (Kovshoff et al. 2017). Although some studies found no age associations (Havermans et al. 2010; Orm et al. 2021), other studies showed that younger siblings (8–11 years) reported more negative adjustment than older siblings (12–16 years), and age was negatively correlated with negative adjustment (Guite et al. 2004; Taylor, Fuggle, and Charman 2001). It is yet unclear if the found differences also represent differences in negative adjustment at the population level, or that the scale measures this construct differently across age groups. We expected the NAS to be invariant across age groups, as this is also the case for general adjustment measures, such as the Strengths and Difficulties Questionnaire (SDQ; Speyer, Auyeung, and Murray 2023) and the Youth Self-Report (YSR; Fonseca-Pedrero et al. 2012).

Third, we investigated the invariance of the scale across two translations, Norwegian and Dutch, to determine if the scale measures the same construct in both languages. Invariance across translations is relevant because translating a scale can impact the psychometric properties. For example, translation procedures can result in ambiguous items, or different item meanings across translations (Ozolins et al. 2020). In addition, cultural differences may influence siblings’ experiences and interpretations (Kovshoff et al. 2017). We hypothesized that the NAS would be invariant across the translations, as the Norwegian and Dutch language and culture show numerous similarities and the used translation procedures were similar (forward and back translation).

Fourth, we studied the internal consistency and factor structure of the parent proxy report version of the NAS, and its correlation with the child report version. The parent report version of the NAS has been used less frequently than the child version and little is known about its psychometric properties (Gutierrez, Horan, and Limbers 2023). Studies comparing the parent and child report NAS, found that parents and children report sibling adjustment differently, and that the versions showed low correlations (Guite et al. 2004; Taylor, Fuggle, and Charman 2001). We therefore hypothesized that there would be differences and a low correlation between the parent report and child report NAS in the current sample, but that the parent version still has good psychometric properties.

## Materials and methods

### Procedures and subsamples

This study was preregistered on OSF.io (Veerman, Willemen, Orm, et al. 2023). Small changes to the procedures were made based on the findings and recent guidelines. The study used baseline data from three sibling intervention trials. The first trial is a Norwegian open trial (2014–2017) into the parent-sibling intervention ‘SIBS’ (Haukeland et al. 2020). Siblings filled out paper questionnaires individually (>11 years), or with assistance from a research assistant (<11 years). The second trial was a subsequent Norwegian randomized controlled trial (RCT; 2019–2023; NCT04056884) of SIBS (Fjermestad, Silverman, and Vatne 2020). Siblings filled out a set of online questionnaires at home, with assistance from parents if needed. The third trial was a Dutch RCT (2022–2024; NCT05376007) into the serious game ‘Broodles’, which was conducted in both the Netherlands and the Dutch-speaking part of Belgium (Veerman, Willemen, Derks, et al. 2023). Siblings and parents filled out online questionnaires at their homes, with all siblings receiving assistance from an independent research assistant. Participants with no data on the NAS (*n* = 98 Norwegian children; *n* = 1 Dutch parent), or who did not give consent for data sharing in collaborative projects (*n* = 4 Dutch parents), were excluded from this study. Ethics approval and informed consent were obtained in all trials. The Norwegian trials were approved by the Regional Committee for Medical and Health Research Ethics, South East (2011/2514; 2018/2461). The Dutch trial was approved by the Medical Ethics Committee of the University Medical Center Amsterdam, location Vrije Universiteit Amsterdam Medical Center (2021.0740, NL79852.029.21). See the referenced publications for more details about the trials.

### Participants

The total sample comprised 400 Norwegian, Dutch and Belgian children aged 6 to 16 years. The 297 Norwegian children (8–16 years) had a sibling with a broad range of disabilities, including rare genetic disorders (34%), autism spectrum disorder (26%), attention-deficit hyperactivity disorder (22%), Down syndrome (8%), congenital heart disease (6%), cerebral palsy (3%), or other (1%). Just over half (55%) had an intellectual disability, of which 33% was explicitly reported, and 22% was estimated based on analysis of the reported diagnoses. The 103 children (6–9 years) in the Dutch sample had a sibling with an intellectual disability (64%), visual impairment (17%) or visual-and-intellectual disability (19%). Some had an additional physical disability (46%), or other additional disorders, such as autism spectrum disorder (27%). In addition, 102 parents participated in the Dutch study. Of these families 16 lived in Belgium and 87 lived in the Netherlands. Based upon parent report at enrolment, the included children and parents did not have a disability themselves, although this was not measured. See Table 1 for an overview of the demographics of the sample.

**Table 1.** Demographics of the Norwegian and Dutch sample.

	Norwegian (*n* = 297)		Dutch (*n* = 103)	
Characteristic	%	M (SD)	%	M (SD)
Age sibling		10.75 (2.11)		7.48 (1.04)
Age parent		*N/A*		41.19 (4.58)
Age child disability		10.50 (3.80)		8.23 (3.51)
Birth order (% older)^a^	49.8%		42.7%	
Gender sibling (% girls)	54.1%		54.0%	
Gender parent (% mothers)	*N/A*		89.2%	
Gender child disability (% girls)	37.0%		47.6%	
Household income^b^	61.0%		77.7%	
Education level (% higher)^c^	70.0%		79.6%	
Work status mother (% working)	81.7%		83.0%	
Work status father (% working)	88.5%		96.9%	
Number of siblings (% >1)	57.1%		63.1%	
Single parent household^d^	21.8%		8.7%	

^a^ % siblings that are older than the child with a disability.

^b^ Norwegian sample: % with ‘good’ to ‘very good’ family financial situation. Dutch sample: % with family income above average.

^c^ Using the education level of the participating parent. Higher education is considered education beyond high school.

^d^ % parents that are divorced, separated, not living together, widowed or no other parent is involved.

### Measurements

We used the 18-item child report and parent proxy report versions of the Negative Adjustment Scale (NAS) to measure siblings’ adjustment to having a sibling with a disability (Lobato and Kao 2002). The NAS stems from the 22-item Sibling Perception Questionnaire (Sahler and Carpenter 1989). In this study, Norwegian (Haukeland et al. 2020) and Dutch (Havermans et al. 2015) translations of the 4-point Likert scale (1 = *never* to 4 = *a lot*) version were used. Previous studies found that both translations of the NAS child report had acceptable to good internal consistencies, with a Cronbach’s alpha of 0.69–0.76 for the Norwegian translation (Haukeland et al. 2020), and 0.82 for the Dutch translation (Havermans et al. 2015). In the Dutch study, the word ‘problem’ in the items was replaced by ‘disability’, in order to fit the sample. Also, a visualization with bars of increasing size was added to the Likert scale in the Dutch questionnaire to adapt it to the age of the children (6–9 years) who participated in that study.

As the literature is unclear about the final item set of the NAS, the used items in the different samples were slightly different (Gutierrez, Horan, and Limbers 2023). Specifically, item 9 in the Norwegian trials was ‘I talk to other adults about my brother or sister’s problem’ (hereafter: item 9a), whereas in the Dutch trial this was ‘I wish my parent would spend less time with my brother/sister’ (hereafter: item 9b). In the Dutch study, all 22 SPQ-items were administered, which includes both versions of item 9. Therefore, the analyses with the combined sample could be conducted using item 9a, and the analyses with only the Dutch sample using item 9b.

Abbreviations for the used items are listed in Tables 2 and 3. The full translated questionnaires are available upon request.

**Table 2.** Item endorsement frequencies and item-rest correlations of the Norwegian child report version (*N* = 297).

Item	Item-rest *r*	*Never* (1)	*A little* (2)	*Sometimes* (3)	*A lot* (4)	Missing
1. Forget about sibling’s problem^a^	0.18	38.0%	19.5%	29.6%	12.8%	0
2. Sad about sibling’s problem	0.63	23.6%	30.0%	29.0%	17.5%	0
3. Have too many household chores	0.43	54.9%	27.3%	15.2%	2.7%	0
4. Afraid of sibling’s illness/disability	0.53	47.6%	26.0%	19.6%	6.8%	1
5. Think about sibling’s illness/disability	0.46	14.2%	42.2%	29.4%	14.2%	1
6. Understand parents spend time with the sibling^a^	0.02	4.1%	12.2%	23.3%	60.5%	1
7. Angry about sibling’s illness/disability	0.43	40.1%	25.3%	22.9%	11.8%	0
8. Wish for someone who understands	0.46	22.7%	25.1%	25.1%	27.1%	2
9a. Talk to other adults about sibling’s problem	0.12	40.7%	36.0%	18.5%	4.7%	0
10. Wish could do something about sibling’s illness/disability	0.36	8.8%	19.5%	30.3%	41.4%	0
11. Wonder why/how sibling got illness/disability	0.31	34.6%	27.1%	21.7%	16.6%	2
12. Wish to spend more time with parents	0.46	20.6%	31.8%	28.4%	19.3%	1
13. Worried about getting same illness/disability	0.34	79.1%	9.1%	7.7%	4.0%	0
14. Friends worried about getting illness/disability	0.30	95.2%	2.0%	2.0%	0.7%	3
15. Feel ignored by parents	0.37	78.0%	15.2%	6.8%	0.0%	1
16. Others are more interested in the sibling	0.32	54.0%	26.7%	14.5%	4.7%	1
17. Others care^a^	0.17	3.4%	19.7%	35.4%	41.5%	3
18. Illness/disability changes family activities	0.37	16.6%	23.7%	29.8%	29.8%	2

*Note*. Abbreviations for the used items are listed. Valid percentages reported.

^a^ Non-reversed item frequencies reported. Reversed items are used for the item-rest correlations.

**Table 3.** Item endorsement frequencies and item-rest correlations of the Dutch child and parent report version (*N* = 103/102).

Item	Item-rest *r*	*Never* (1)	*A little* (2)	*Sometimes* (3)	*A lot* (4)
1. Forget about sibling’s disability^a^	0.10/0.19	68.0/33.3%	15.5/26.5%	10.7/25.5%	5.8/14.7%
2. Sad about sibling’s disability	**0.47/0.66**	39.8/17.7%	25.2/21.6%	25.2/49.0%	9.7/11.8%
3. Have too many household chores	0.43/0.39	22.3/32.4%	33.0/36.3%	29.1/27.5%	15.5/3.9%
4. Afraid of sibling’s disability	0.44/0.38	73.8/63.7%	16.5/23.5%	5.8/10.8%	3.8/2.0%
5. Think about sibling’s disability	**0.44/0.53**	21.4/2.0%	33.0/23.5%	25.2/49.0%	20.4/25.5%
6. Understand parents spend time with the sibling^a^	0.07/0.06	7.8/0.0%	10.7/8.8%	24.3/54.9%	57.3/36.3%
7. Angry about sibling’s disability	**0.33/0.57**	65.1/25.5%	18.5/26.5%	6.8/33.3%	9.7/14.7%
8. Wish for someone who understands	**0.33/0.42**	26.2/19.6%	18.5/31.4%	29.1/36.3%	26.2/12.8%
9a. Talk to other adults about sibling’s disability	*N/A*	54.4/13.7%	23.3/50.0%	17.5/30.4%	4.8/5.9%
9b. Wish parents spend less time with the sibling	0.47/0.46	35.0/17.7%	29.1/27.5%	18.5/43.1%	17.5/11.8%
10. Wish could do something about sibling’s disability	**0.34/0.51**	14.6/16.7%	16.5/21.6%	21.4/37.3%	47.6/24.5%
11. Wonder why/how sibling got disability	0.35/0.39	25.2/19.6%	13.6/38.2%	30.1/32.4%	31.1/9.8%
12. Wish to spend more time with parents	0.49/0.42	19.4/4.9%	21.4/17.7%	35.9/45.1%	23.3/32.4%
13. Worried about getting same disability	**0.35/0.25**	80.6/86.3%	6.8/10.8%	2.9/1.0%	9.7/2.0%
14. Friends worried about getting disability	**0.30/0.20**	86.4/92.2%	8.7/7.8%	3.9/0.0%	1.0/0.0%
15. Feel ignored by parents	**0.36/0.51**	44.7/16.7%	20.4/40.2%	27.2/33.3%	7.8/9.8%
16. Others are more interested in the sibling	0.37/0.30	19.4/22.6%	36.9/30.4%	25.2/31.4%	18.5/15.7%
17. Others care/are concerned^a^	**0.38/0.12**	52.4/12.8%	25.2/31.4%	18.5/45.1%	3.9/10.8%
18. Disability changes family activities	**0.21/0.44**	21.4/1.0%	30.1/8.8%	27.2/38.2%	21.4/52.0%

*Note*. Abbreviations for the used items are listed. Reported as child/parent version. Item-rest correlations are based on the version with item 9b. Differences in item-rest correlations of ≥0.10 are indicated with bold face.

^a^ Non-reversed item frequencies reported. Reversed items are used for the item-rest correlations.

### Data analyses

Using semPower (Moshagen and Bader 2024), an a priori power calculation (*α* = 0.05, *df* = 135, *p* = 18) indicated a sample of 139 participants per group was needed to have sufficient power (0.8) to detect good model fit (Root Mean Square Error of Approximation (RMSEA) = 0.05). To detect acceptable model fit (RMSEA = 0.08), a sample size of 55 participants per group was sufficient, which was the case in all the current analyses.

IBM SPSS Statistics version 28 was used to explore the data (IBM Corp 2021). Outliers were explored based on their inter quartile range (IQR), but not winsorized. Missing data on the item level was present in the Norwegian open trial dataset only (0.9% of all values, see Table 2). Given the low amount of missing data, pairwise deletion was used (Shi et al. 2020).

JASP version 0.18 was used to analyze the data (JASP Team 2024). The descriptives were analyzed in the subsamples. To assess invariance, we first ran regular confirmatory factor analyses (CFAs), testing the previously found single-factor model using Diagonally Weighted Least Squared (DWLS) estimation in the Lavaan package (Park 2023). The goodness of fit was assessed using the Chi-Square Test of Model Fit (χ^2^), RMSEA, Comparative Fit Index (CFI), Tucker–Lewis index (TLI), and standardized root mean square residual (SRMR). To determine an acceptable fit, we used the cut-off scores as reported by Schweizer (2010). For χ^2^ to degrees of freedom ratio (χ^2^/df) scores >3 were considered ‘poor’, <3 ‘acceptable’, and <2 ‘good’. For RMSEA scores > 0.08 were considered ‘poor’, <0.08 ‘acceptable, and <0.05 ‘good’. For CFI and TLI scores <0.90 were considered ‘poor’, >0.90 ‘acceptable’, and >0.95 ‘good’. SRMR scores needed to be <0.10 to be considered ‘acceptable’ and <0.08 to be considered ‘good’.

Next, we ran a multi-group confirmatory factor analysis (MG-CFA) to assess measurement invariance across age groups (1 = 8–11 years, 2 = 12–16 years) in the Norwegian sample, as this is the largest sample with the widest age range. We used item 9a in this analysis, as this item was included in the total sample. A stepwise procedure, as described by Leitgöb et al. (2023), was used. We first assessed configural invariance, testing if the single factor structure, with all items measuring one latent construct, holds in both groups. Next, we assessed metric invariance, testing if the factor loadings of the items are equal in both groups. Finally, we assessed scalar invariance, testing if the intercepts of the items are equal in both groups. At each step, we evaluated if the model fit of the more restricted model did not significantly deteriorate, by considering change in Chi-Square (which should not be significant) and CFI (which should be no more than 0.01). The same invariance analyses were conducted to assess measurement invariance across translations (1 = Norwegian, 2 = Dutch). Based on the results of the invariance analysis across age, we determined if we could use the total Norwegian sample or only those in the age group 8–11 years.

Cronbach’s alphas and McDonald’s omegas were calculated in separate samples and the total sample for the set of items with the best fitting factor solution. We evaluated the internal consistency using the cut-off scores as described by George and Mallery (2003).

Finally, the factor structure of the parent and child version, including item 9b, was analyzed in the Dutch sample with a CFA of the one-factor model. The Pearson correlation with the child report version was calculated, and differences in item statistics between the parent and child version were visually inspected.

## Results

### Child report version

#### Descriptives

Inspection of outliers showed three mild outliers (1.5 × IQR; item 4, 6, 7) and two extreme outliers (3 x IQR; item 13, 14) in the total dataset. Endorsement frequencies, inter-item correlations, and item-rest correlations in the total Norwegian, and Dutch sample and the Norwegian subsamples of the two age groups were inspected (see Tables 2 and 3, and Supplementary Appendix 1, Table S1–S4). Floor effects were present on item 13 (worried about getting same illness/disability) and item 14 (friends worried about getting illness/disability), as ≥80% of the participants scored ‘never’ (1) on these items.

**Table 4.** Summary of the measurement invariance analyses across age groups and translations.

	χ²	*df*	*p*	χ²/*df*	CFI	TLI	RMSEA	SRMR	Δχ²	Δ*df*	ΔCFI
**Across age groups** (*N* = 297)											
Configural (16-item)	297.36	208	<.001	1.43	0.96	0.96	0.054	0.098			
Configural (13-item)	184.20	130	.001	1.42	0.98	0.97	0.053	0.092			
Metric	221.84	142	<.001	1.56	0.96	0.96	0.062	0.101	37.64**	12	0.012
**Across translations** (*N* = 299)											
Configural (16-item)	306.57	208	<.001	1.47	0.95	0.94	0.056	0.105			
Configural (14-item)	224.23	154	<.001	1.46	0.96	0.96	0.055	0.101			
Metric	247.20	167	<.001	1.48	0.96	0.95	0.057	0.106	22.97*	13	0.006
Scalar	468.83	193	<.001	2.43	0.85	0.86	0.098	0.109	221.63**	26	0.112

* *p* < .05; **p < .001.

#### Measurement invariance across age groups

In the Norwegian sample (*N* = 297), a CFA including all 18 items (using item 9a) showed good model fit of the one-factor model on most indices (*χ^2^* (135) = 246.92, *p* < .001, *χ^2^/df* = 1.83, CFI = 0.95, TLI = 0.95, RMSEA = 0.053, SRMR = 0.085). The factor loading of item 6 was not significant (*λ* = 0.023, *p* = .493) and was thus removed. The model fit remained similar (*χ^2^* (119) = 218.82, *p* < .001, *χ^2^/df* = 1.84, CFI = 0.96, TLI = 0.95, RMSEA = 0.053, SRMR = 0.085).

To run the MG-CFA, it was necessary to remove item 14 from the model, as this variable caused an error in the model, even when merging 4-scores with 3-scores in one of the groups and imputing the missing data, possibly because of a floor effect (>95% score 1). The 16-item configural model had acceptable fit (see Table 4). However, the factor loadings on item 9a (λ = 0.237; 0.079) and 17 (λ = 0.158; 0.253) were unacceptable in both groups (8–11 years; 12–16 years), and loadings on item 1 were unacceptable in the group with age 8–11 years (*λ* = 0.09). Therefore, these items were removed from the model. The final 13-item model (excluding items 1, 6, 9a, 14 and 17) had a slightly improved fit. A significant difference (confidence intervals do not overlap) in factor loadings was present on item 7 (see Table 5).

**Table 5.** Factor loadings and residual variances in the subsamples.

	Standardized factor loading				Residual variance	
Item	8–11 years (*n* = 196)	95% *C.I.*	12–16 years (*n* = 101)	95% *C.I.*	8–11 years	12–16 years
Age groups						
NAS2	0.804	0.726–0.882	0.797	0.698–0.897	0.354	0.364
NAS3	0.510	0.426–0.594	0.672	0.573–0.771	0.740	0.548
NAS4	0.691	0.613–0.770	0.726	0.628–0.824	0.522	0.473
NAS5	0.560	0.486–0.633	0.665	0.569–0.761	0.687	0.558
NAS7	0.421	0.343–0.498	0.730	0.633–0.826	0.823	0.468
NAS8	0.496	0.421–0.572	0.635	0.537–0.734	0.754	0.596
NAS10	0.535	0.457–0.614	0.475	0.378–0.572	0.713	0.775
NAS11	0.386	0.306–0.466	0.372	0.272–0.472	0.851	0.862
NAS12	0.556	0.478–0.633	0.552	0.454–0.650	0.691	0.696
NAS13	0.552	0.458–0.645	0.509	0.390–0.628	0.696	0.741
NAS15	0.617	0.511–0.722	0.615	0.501–0.728	0.620	0.622
NAS16	0.474	0.390–0.559	0.284	0.173–0.395	0.775	0.919
NAS18	0.465	0.388–0.541	0.520	0.418–0.623	0.784	0.729
Translations	Norwegian *(n* = 196)	95% *C.I.*	Dutch (*n* = 103)	95% *C.I.*	Norwegian	Dutch
NAS2	0.799	0.723–0.876	0.602	0.482–0.723	0.361	0.637
NAS3	0.511	0.428–0.594	0.570	0.454–0.686	0.739	0.675
NAS4	0.685	0.608–0.762	0.660	0.529–0.791	0.531	0.565
NAS5	0.560	0.487–0.632	0.568	0.448–0.688	0.687	0.678
NAS7	0.438	0.361–0.514	0.534	0.383–0.645	0.808	0.736
NAS8	0.496	0.421–0.571	0.421	0.305–0.538	0.754	0.822
NAS10	0.539	0.462–0.616	0.435	0.313–0.558	0.709	0.811
NAS11	0.393	0.314–0.471	0.405	0.285–0.525	0.846	0.836
NAS12	0.559	0.482–0.635	0.611	0.493–0.729	0.688	0.626
NAS13	0.552	0.461–0.644	0.526	0.370–0.681	0.695	0.724
NAS14	0.679	0.527–0.831	0.331	0.186–0.477	0.539	0.890
NAS15	0.600	0.498–0.702	0.478	0.346–0.610	0.640	0.772
NAS16	0.467	0.384–0.549	0.433	0.319–0.546	0.782	0.813
NAS18	0.471	0.395–0.547	0.327	0.216–0.439	0.778	0.893

*Note*. Different items are included in the models across the analyses.

Next, the metric model was assessed. The model fit deteriorated significantly on both fit indices (see Table 4). The null hypothesis of metric invariance was therefore rejected, and consequently scalar invariance was not assessed.

#### Measurement invariance across translations

Because the NAS did not show invariance across age groups, we assessed invariance across translations in a combined sample (*n* = 299) of only the Norwegian children aged 8–11 years old, and the Dutch children. Using all 18 items (including item 9a), the one-factor model showed poor model fit (*χ^2^* (135) = 456.66, *p* < .001, *χ^2^/df* = 3.38, CFI = 0.83, TLI = 0.81, RMSEA = 0.089, SRMR = 0.108). When removing items 6 (understand parents spend time with the sibling) and 17 (others care/are concerned) from the model, which had non-significant factor loadings, the model showed acceptable fit on most of the indices (*χ^2^* (104) = 283.87, *p* < .001, *χ^2^/df* = 2.73, CFI = 0.90, TLI = 0.88, RMSEA = 0.076, SRMR = 0.097). The poor fit of these items makes sense, as this includes positively formulated items that might not cover the same construct when reversed.

To run the MG-CFA, it was necessary to merge 4-scores with 3-scores on item 15 for 8 cases (7.8%) in the Dutch sample, so the categories were uniform across the two groups. This was because no participant in the Norwegian samples had reported a score of 4 on this item. The 16-item model had good fit on most indices. Factor loadings were generally lower in the Dutch dataset, and thus residual variances were larger (see Table 5). Confidence intervals of the factor loadings did not overlap across groups for item 14, indicating a significant difference (see Table 5). Item 1 (forget about sibling’s problem/disability; *λ* = 0.079; 0.149) and 9a (talk to other adults about sibling’s problem/disability; *λ* = 0.245; 0.239) were removed, as the factor loadings were below 0.30 in both samples (Norwegian; Dutch), which is not acceptable (Brown 2015). The poor fit of these items might be related to differences in item meaning, because of the use of the word ‘problem’ in the Norwegian version, versus ‘disability’ in the Dutch version. The poor fit might also be related to the fact that item 1 is a reversed item, and item 9a belonged to the communication subscale in the original SPQ. The final 14-item configural model (excluding items 1, 6, 9a and 17) showed an acceptable to good model fit on most indices (see Table 4).

Next, metric invariance was tested by constraining the factor loadings across groups. Model fit deteriorated, with a significant Chi-square difference test (*p* = .042), but a CFI decrease smaller than 0.01. Thus, the indices are inconclusive regarding metric invariance, and therefore scalar invariance was tested next, adding restrictions to the intercepts as well. The scalar model, however, showed a significant deterioration in model fit, with a significant Chi-square difference test, and a CFI decrease larger than 0.01 (see Table 4). Scalar invariance was therefore not supported, which implies that latent means cannot be compared between the groups (Leitgöb et al. 2023).

#### Internal consistency

Internal consistency was analysed using the model with the best fit and adequate factor loadings. The 14-item child report scale (excluding item 1, 6, 9 and 17) showed acceptable to good internal consistency in the combined sample of children aged 6–11 years (*α* = 0.76, *ω* = 0.77), Norwegian total sample (*α* = 0.80, *ω* = 0.78), and Dutch sample (*α* = 0.75, *ω* = 0.75). In the Norwegian subsample of children aged 8–11 years the internal consistency was acceptable to good (*α* = 0.79, *ω* = 0.79). In the subsample of children aged 12–16 years the internal consistency was good (*α* = 0.82, *ω* = 0.83).

### Parent proxy report versus child report version

Inspection of outliers showed four mild outliers (item 4, 5, 12, 18) and two extreme outliers (item 13, 14) in the parent data. Item 13 and item 14 exhibited comparable floor effects to those seen in the child report version. Endorsement frequencies, inter-item correlations, and item-rest correlations were examined (see Table 3 and Supplementary Appendix 1, Table S3).

Comparing the parent with the child report scale, numerous differences in item characteristics were found (see Table 3). More than half of the items showed a difference of ≥0.10 in item-rest correlations. For example, item 17 (other’s care/are concerned) fits in better with the other items in the child report scale than in the parent report scale. This might be explained by the ambiguity of the item phrasing, that might be more confusing to adults. Differences in endorsement frequencies showed that parents perceive the experiences of their child different than the child does themselves. For example, parents indicate that their child experiences more anger about the disability than children do themselves.

We evaluated the fit of the one-factor model of the parent version using a CFA, with item 9b utilized due to its stronger inter-item correlations compared to item 9a. Item 17 (others care/are concerned) was not reversed, as the item did not correlate negatively with the other items in this sample. The 18-item model showed poor fit (see Table 6). All factor loadings were significant, but <0.20 on item 6 and 17 (*λ* = 0.108; 0.145). When these items were removed, the model fit slightly improved, but still showed poor model fit on part of the indices. Based on the modification indices (MI = 38.86; 24.43), residual covariances between item 12 (wish to spend more time with parents) and 15 (feel ignored by parents) and item 9b (wish parents spend less time with the sibling) and 12 were added, which resulted in an acceptable model fit on most of the indices (see Table 6). The factor loadings of the 16 items were acceptable (see Table 7).

**Table 6.** Summary of the confirmatory factor analyses in the Dutch parent and child report versions.

	χ²	*df*	*p*	χ²/*df*	CFI	TLI	RMSEA	SRMR
**Parent report** (*N* = 102)								
18-item model	299.28	135	<.001	2.22	0.89	0.87	0.110	0.141
16-item model^a^	231.76	104	<.001	2.23	0.91	0.90	0.110	0.134
16-item model, cov.^b^	148.93	102	<.001	1.46	0.97	0.96	0.067	0.116
**Child report** (*N* = 103)								
18 item model	197.35	135	<.001	1.46	0.92	0.91	0.067	0.121
16-item model^c^	149.88	104	.002	1.44	0.94	0.93	0.066	0.116

^a^ Items 6 and 17 omitted.

^b^ Covariance added between item 12 and 15, and between item 9b and 12.

^c^ Items 1 and 6 omitted.

**Table 7.** Factor loadings and residual variances of the parent report version.

Item	Factor Loading	95% *C.I.*	Residual Variance
NAS1^a^	0.315	0.217–0.413	0.901
NAS2	0.835	0.745–0.926	0.302
NAS3	0.534	0.444–0.624	0.715
NAS4	0.522	0.419–0.624	0.728
NAS5	0.675	0.594–0.757	0.544
NAS7	0.683	0.595–0.772	0.533
NAS8	0.518	0.421–0.614	0.732
NAS9b	0.546	0.453–0.638	0.702
NAS10	0.692	0.607–0.776	0.522
NAS11	0.497	0.403–0.591	0.753
NAS12	0.337	0.235–0.438	0.887
NAS13	0.450	0.308–0.592	0.798
NAS14	0.368	0.167–0.569	0.865
NAS15	0.513	0.419–0.606	0.737
NAS16	0.352	0.254–0.450	0.876
NAS18	0.568	0.461–0.676	0.677

*Note*. Standardized factor loadings are reported.

^a^ Reversed items.

To inspect the differences in factor loadings and fit between the parent and child version, we also ran the CFA in the Dutch child sample. The confidence intervals of the factor loadings did not overlap across informants for items 2, 10, 17 and 18, indicating significant differences (see Supplementary Appendix 1, Table S5). In both samples, item 6 was omitted. In the parent sample, item 17 was omitted, but in the child sample item 1 was omitted, based on factor loadings. In the child version, no post hoc modifications were needed to reach an acceptable fit on most of the indices (see Table 6).

The internal consistency of the 16-item parent scale was good, with a Cronbach’s alpha of 0.81 and a McDonald’s omega of 0.82. The 16-item child scale showed acceptable to good internal consistency (*α* = 0.78, *ω* = 0.79). The parent report version of the total 16-item scale did not significantly correlate with the child report version of the scale (*r* = 0.16, *p* = .113).

**Figure 1.**
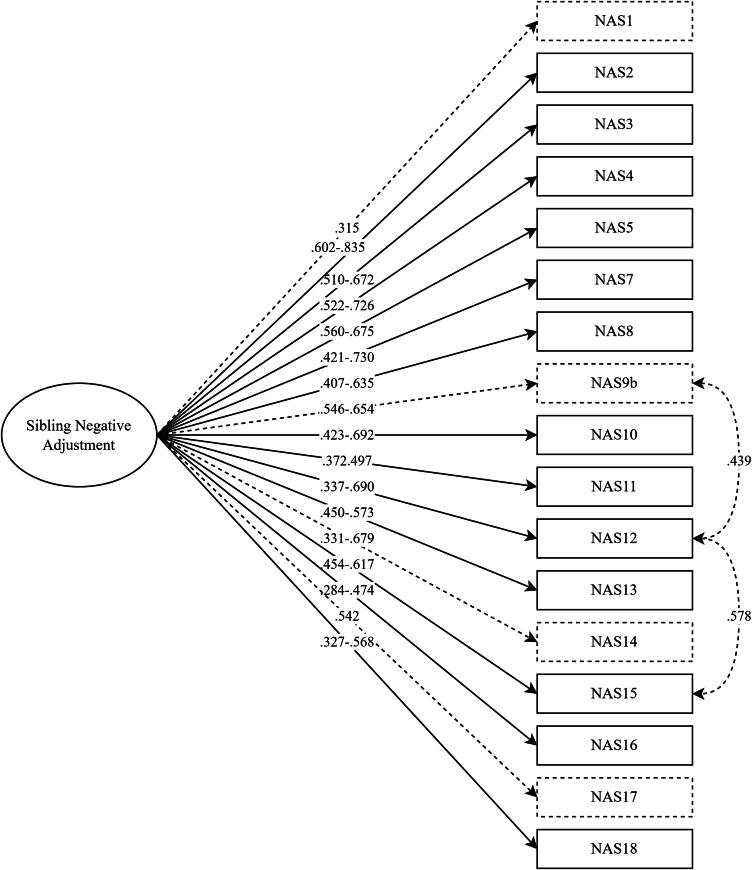
Factor model of the NAS across versions and groups. *Note*. The lowest and highest found factor loadings of the final models, across the versions and groups, are reported. Dashed lines and boxes indicate items that only showed acceptable factor loadings in part of the versions and groups. The covariances are only applicable to the parent version.

Figure 1 shows the factor structure of the NAS and gives a summary of the results across all computed factor analyses.

## Discussion

The aim of this study was to examine the psychometric properties of the NAS across different translations, age groups and versions (child and parent report). Previous studies showed that the NAS generally has promising psychometric properties (Gutierrez, Horan, and Limbers 2023; Orm et al. 2021). The current study partly confirmed this, but also showed that modifications are required to use the NAS in heterogenous samples of siblings of children with disabilities, and in comparative studies.

The main findings were first, that we confirmed the single-factor structure and acceptable internal consistency that was previously found for the NAS (Orm et al. 2021). Furthermore, this single-factor structure held across all subsamples and versions. However, the NAS did not show higher level invariance across age groups (8–11 years versus 12–16 years). Across translations (Norwegian versus Dutch), fit indices were inconclusive about metric invariance, meaning that the pattern and weight of the factor loadings were not equal. Nevertheless, scalar invariance was not supported, meaning that the intercepts were not equal. As a result, the means cannot validly be compared between these age groups and translations (Leitgöb et al. 2023).

Finally, the parent proxy report version showed acceptable internal consistency and fit of the single-factor model. However, it showed a poorer model fit than the child report version, and more post hoc adaptations were needed to reach an acceptable model fit. The parent report version showed a low and non-significant correlation with the child report version, and numerous differences in item characteristics, which is in line with previous studies (Guite et al. 2004).

### Study limitations

The current study has a few limitations, that might partially explain why the hypothesized invariance across age groups and translations were not found. First, potential differences in other background variables, such as type of disability, might have impacted the results, as this can influence the experiences of siblings and thus the interpretation of items (Wolff et al. 2022). This is also the case for a wider range of variables, that we did not measure, such as siblings’ own disability-related traits (Wolff, Franco, et al. 2023) or other influential factors on the family or society level (Kovshoff et al. 2017), such as parental stress and social support. This could have impacted the variance in the Norwegian and Dutch samples in different ways, which possibly explains why invariance was not confirmed.

Second, in the assessment of invariance across translations, age differences between the Norwegian and Dutch sample may have impacted the results. Although we minimized the age difference by only including the children aged 8–11 years from the Norwegian sample, the children in the Dutch sample were still overall younger (6–9 years). Even though children from the age of 6 can validly complete questionnaires about their health and psychological functioning, reliability and validity generally increase with age (Mellor 2004; Riley 2004). Differences in how the NAS was administered across studies might have affected the outcomes as well, i.e. paper or online questionnaires, provided assistance, and the use of visualization of the response categories (Bowling 2005). The model fit was somewhat better in the Norwegian than in the Dutch version, possibly because additional adaptations were made to the Dutch translation. For example, by replacing the word ‘problem’ with ‘disability’ in some of the items, the meaning of the items slightly changed. Similarly, the results may have been affected by translations being slightly different on some items.

As for the parent report scale, the young age of the siblings (6–9 years) in our Dutch subsample might have influenced the results, since the younger age of siblings could be related to higher discordance between child and parent report (Guite et al. 2004). Also, whereas parents completed the questionnaire independently, children completed the questionnaire with assistance, which might have influenced their responses to be more socially desirable (Kooijmans, Langdon, and Moonen 2022).

Despite these limitations, this study profited from sufficient sample sizes, comparable item sets, and variance in response tendencies. Also, it is the first to assess the invariance of the NAS (Gutierrez, Horan, and Limbers 2023). The finding that the unifactorial structure of the NAS holds across different translations and heterogenous samples (in terms of age and disabilities) and with acceptable internal consistency is important, because sibling studies often include children with a wide range of ages and sibling diagnoses (Wolff, Magiati, et al. 2023). The results suggests that the NAS could be promising to further develop into a psychometrically sound measure of sibling adjustment across different contexts.

Still, the current study investigated the NAS across two cultures that are quite similar and both more individualistic than collectivistic, and this study does not provide insight into the invariance across more diverse cultures. The scale might show a different factor structure in cultures that are more collectivist, such as Taiwan, as social norms, stigma and cultural values can influence siblings’ experiences or how they report these (Tsai et al. 2018).

### Adaptations to the scale

Although the scale has acceptable to good internal consistency and factor structure across versions, adjustments are needed, and several items appear problematic. This slightly varies across versions, but in general it seems that the (reversed) positively phrased items (1, 6, 9a and 17) do not consistently fit well within the negative adjustment scale. This suggests that these items measure another aspect of sibling adjustment, that does not capture negative adjustment when reversed. Moreover, item 9a (talk to other adults about sibling’s problem/disability), that was originally part of the Communication subscale of the SPQ, does not seem to fit well in the NAS (Lobato and Kao 2002). Possibly, these items could be part of a positive adjustment subscale, with new additional items. As suggested by Orm et al. (2021), it is desirable to develop such a subscale, to capture both the positive and negative aspects of the sibling experience. Sibling research has been criticized for its invalid focus on the negative effects of living with a sibling with a disability, although studies have showed that siblings also experience positive effects (Hayden and Hastings 2022; Hastings 2016). The negative focus of the NAS adds to this biased view and does not fit in with the current paradigm. Also, from the viewpoint of the Siblings Embedded Systems Framework (Kovshoff et al. 2017), the scale does not fully capture the systemic nature of sibling adjustment. For example, items about societal aspects, such as stigma are missing.

In addition, the one-on-one adaptation from the items which were originally meant to capture the experiences of siblings of children with cancer, to a scale for siblings of children with a variety of diagnoses, might be problematic. Inspection of the NAS-items shows that not all items seem to fit well with the experiences of siblings of children with developmental disabilities. For example, items 13 and 14, measuring the fear of developing the same disability as their sibling, showed pronounced floor effects. This makes sense, as unlike siblings of children with cancer, siblings of children with disabilities might not have such fear in the same degree. More relevant for this target group is to include items based on qualitative sibling studies that reflect the experiences of siblings of children with disabilities, for example about fear of aggression or worry about the future (Moyson and Roeyers 2012; Schumann, Vatne, and Fjermestad 2024).

Finally, for the parent version of the scale, the single-factor structure of the NAS might not be optimal. It should be considered that the parent scale may comprise multiple factors, as a recent study found a better fit for a four-factor model of the parent-version of the total SPQ (Gutierrez and Limbers 2024). A more complex factor structure for the parent version makes conceptual sense, since the questionnaire includes items about interpersonal and intrapersonal aspects of sibling adjustment. These aspects may be highly intertwined for siblings themselves (Orm et al. 2021), but be more separatable for parents. However, in general, sibling negative adjustment has not been clearly defined, and as the SPQ originally measured siblings’ perceptions in a broader sense, its conceptualization might be flawed.

### Implications and future directions

The current study has implications for research and practice. Our findings underline the need for assessing invariance, to prevent biased results when using questionnaires in large and diverse, or combined samples or making comparisons between groups. Careful consideration is required by researchers when translating and adapting questionnaires (Ozolins et al. 2020), as it might impact the psychometric properties.

This is also the case for differences in age of participating children. Researchers should be cautious in using the NAS to make comparisons between age groups and consider bias when using it in a sample with a large age range (i.e. school age children and teenagers). The previously found negative association between NAS scores and age (Taylor, Fuggle, and Charman 2001; Guite et al. 2004), might be caused by a change in the underlying dimension of negative adjustment when children grow older. On the item level, some items appeared more meaningful to younger children compared to older children, or vice versa. Some items may capture aspects of negative adjustment that are specific to children in a certain age group. In addition, researchers and clinicians need to be thoughtful when choosing the informant to measure sibling adjustment that best fits their research question, as both versions provide different insights.

In order to further assess the usability of the NAS, future research could focus on investigating which items can validly be used in comparative studies and diverse samples. For example, difference in item functioning could be assessed with Item Response Analysis (Stark, Chernyshenko, and Drasgow 2006). It could also be beneficial to assess partial invariance, to determine the items that are problematic and select a subset of items that are invariant across groups (Pokropek, Davidov, and Schmidt 2019). Validation of the scale in other cultures and languages, with consideration of other relevant variables, such as the diagnosis of the child and the sibling’s own developmental functioning, is needed as well.

In addition, it is of importance to explore and enhance the clinical usability of the scale in identifying the individual intervention needs of siblings. This is necessary, as siblings find it hard to phrase their support needs at a young age (Hanvey, Malovic, and Ntontis 2022), and often only receive support when problems arise (Bergvoll et al. 2023). Early identification of support needs could promote adequate support and prevent psychological problems that siblings can experience (Martinez et al. 2022).

## Conclusion

In conclusion, this study provides evidence that an adapted version of the NAS, omitting items that show poor fit, can be considered as a sibling-tailored outcome measure. However, the scale is less suitable to make comparisons between samples and subgroups, and bias needs to be considered when using it in a diverse sample regarding language and age. Moving forward, researchers should either adapt and further validate the NAS, consider and investigate other sibling-tailored scales, or develop a new scale based on existing scales, that better captures the complex nature of sibling adjustment. This measure needs to be appropriate to use across different age groups, cultures and types of diagnoses. An international collaborative group of sibling researchers should be involved in this further process. Moreover, the engagement of clinicians and siblings of children with disabilities is crucial for application in clinical practice.

## Supplementary Material

Supplemental Material

## Data Availability

The data that support the findings of this study are available from the corresponding author, Linda K. M. Veerman, upon reasonable request.

## References

[CIT0001] Bergvoll, L.‐M., S. S. Fjelldal, A. Clancy, M. Martinussen, and H. Laholt. 2023. “How Do Public Health Nurses in Norwegian School Health Services Support Siblings of Children with Complex Care Needs?” *Scandinavian Journal of Caring Sciences* 37 (4): 1100–1108. 10.1111/scs.13184. 37246570

[CIT0002] Blamires, J., M. Foster, S. Rasmussen, M. Zgambo, and E. Mörelius. 2024. “The Experiences and Perceptions of Healthy Siblings of Children with a Long-Term Condition: Umbrella Review.” *Journal of Pediatric Nursing* 77: 191–203. 10.1016/j.pedn.2024.03.022. 38574402

[CIT0003] Bowling, A. 2005. “Mode of Questionnaire Administration Can Have Serious Effects on Data Quality.” *Journal of Public Health* 27 (3): 281–291. 10.1093/pubmed/fdi031. 15870099

[CIT0004] Brown, T. A. 2015. *Confirmatory Factor Analysis for Applied Research*. 2nd ed. New York: Guilford Press.

[CIT0504] Eccles, Jacquelynne S. 1999. “The Development of Children Ages 6 to 14.” *The Future of Children* 9 (2): 30–44. 10.2307/1602703. 10646256

[CIT0006] Fonseca-Pedrero, E., S. Sierra-Baigrie, S. Lemos-Giráldez, M. Paino, and J. Muñiz. 2012. “Dimensional Structure and Measurement Invariance of the Youth Self-Report Across Gender and Age.” *The Journal of Adolescent Health* 50 (2): 148–153. 10.1016/j.jadohealth.2011.05.011. 22265110

[CIT0007] Fredriksen, T., T. M. Vatne, Y. B. Haukeland, M. Tudor, and K. W. Fjermestad. 2021. “Siblings of Children with Chronic Disorders: Family and Relational Factors as Predictors of Mental Health.” *Journal of Child Health Care* 27 (1): 145–159. 10.1177/13674935211052157. 34727780

[CIT0008] George, D., and P. Mallery. 2003. *SPSS for Windows Step by Step: A Simple Guide and Reference. 11.0 Update*. 4th ed. Boston: Allyn & Bacon.

[CIT0009] Goodman, R. 1997. “The Strengths and Difficulties Questionnaire: A Research Note.” *Journal of Child Psychology and Psychiatry, and Allied Disciplines* 38 (5): 581–586. 10.1111/j.1469-7610.1997.tb01545.x. 9255702

[CIT0010] Guite, J., D. Lobato, B. Kao, and W. Plante. 2004. “Discordance Between Sibling and Parent Reports of the Impact of Chronic Illness and Disability on Siblings.” *Children’s Health Care* 33 (1): 77–92. 10.1207/s15326888chc3301_5.

[CIT0011] Gutierrez, A. L., and C. A. Limbers. 2024. “Validation of the Sibling Perception Questionnaire in Healthy Siblings of Children with Chronic Illnesses.” *Journal of Health Psychology* 0 (0): 13591053241235095. 10.1177/13591053241235095. 38485711

[CIT0012] Gutierrez, A., M. Horan, and C. A. Limbers. 2023. “A Systematic Review of the Psychometric Properties of the Sibling Perception Questionnaire.” *Clinical Child Psychology and Psychiatry* 28 (3): 1192–1216. 10.1177/13591045231157141. 36878184

[CIT0013] Hanvey, I., A. Malovic, and E. Ntontis. 2022. “Glass Children: The Lived Experiences of Siblings of People with a Disability or Chronic Illness.” *Journal of Community & Applied Social Psychology* 32 (5): 936–948. 10.1002/casp.2602.

[CIT0014] Hartling, L., A. Milne, L. Tjosvold, D. Wrightson, J. Gallivan, and A. S. Newton. 2014. “A Systematic Review of Interventions to Support Siblings of Children with Chronic Illness or Disability.” *Journal of Paediatrics and Child Health* 50 (10): E26–E38. 10.1111/j.1440-1754.2010.01771.x. 20598075

[CIT0015] Hastings, R. P. 2016. “Do Children With Intellectual and Developmental Disabilities Have a Negative Impact on Other Family Members? The Case for Rejecting a Negative Narrative.” *International Review of Research in Developmental Disabilities* 50: 165–194. 10.1016/bs.irrdd.2016.05.002.

[CIT0016] Haukeland, Y. B., K. W. Fjermestad, S. Mossige, and T. M. Vatne. 2015. “Emotional Experiences Among Siblings of Children with Rare Disorders.” *Journal of Pediatric Psychology* 40 (7): 712–720. 10.1093/jpepsy/jsv022. 25817880

[CIT0017] Haukeland, Y. B., N. O. Czajkowski, K. W. Fjermestad, W. K. Silverman, S. Mossige, and T. M. Vatne. 2020. “Evaluation of ‘SIBS’, An Intervention for Siblings and Parents of Children with Chronic Disorders.” *Journal of Child and Family Studies* 29 (8): 2201–2217. 10.1007/s10826-020-01737-x.

[CIT0018] Havermans, T., I. De Croock, T. Vercruysse, E. Goethals, and I. Van Diest. 2015. “Belgian Siblings of Children with a Chronic Illness: Is Their Quality of Life Different from Their Peers?” *Journal of Child Health Care* 19 (2): 154–166. 10.1177/1367493513503582. 24154844

[CIT0019] Havermans, T., L. Wuytack, J. Deboel, A. Tijtgat, A. Malfroot, C. De Boeck, and M. Proesmans. 2010. “Siblings of Children with Cystic Fibrosis: Quality of Life and the Impact of Illness.” *Child: Care, Health and Development* 37 (2): 252–260. 10.1111/j.1365-2214.2010.01165.x. 21083689

[CIT0020] Hayden, N. K., and R. P. Hastings. 2022. “Family Theories and Siblings of People with Intellectual and Developmental Disabilities.” *International Review of Research in Developmental Disabilities* 63: 1–49. 10.1016/bs.irrdd.2022.09.001.

[CIT0021] IBM Corp. 2021. “IBM SPSS Statistics for Windows. V. 28.0.” IBM Corp. https://www.ibm.com/products/spss-statistics.

[CIT0022] JASP Team. 2024. “JASP. V. 0.18.” JASP Team. https://jasp-stats.org/.

[CIT0023] Kooijmans, R., P. E. Langdon, and X. Moonen. 2022. “Assisting Children and Youth with Completing Self-Report Instruments Introduces Bias: A Mixed-Method Study That Includes Children and Young People’s Views.” *Methods in Psychology* 7: 100102. 10.1016/j.metip.2022.100102.

[CIT0024] Kovshoff, H., K. Cebula, H.-W. J. Tsai, and R. P. Hastings. 2017. “Siblings of Children with Autism: The Siblings Embedded Systems Framework.” *Current Developmental Disorders Reports* 4 (2): 37–45. 10.1007/s40474-017-0110-5. 28680793 PMC5488140

[CIT0025] Leedham, A. T., A. R. Thompson, and M. Freeth. 2020. “A Thematic Synthesis of Siblings’ Lived Experiences of Autism: Distress, Responsibilities, Compassion and Connection.” *Research in Developmental Disabilities* 97: 103547. 10.1016/j.ridd.2019.103547. 31869772

[CIT0026] Leitgöb, H., D. Seddig, T. Asparouhov, D. Behr, E. Davidov, K. De Roover, S. Jak, et al. 2023. “Measurement Invariance in the Social Sciences: Historical Development, Methodological Challenges, State of the Art, and Future Perspectives.” *Social Science Research* 110: 102805. 10.1016/j.ssresearch.2022.102805. 36796989

[CIT0027] Lobato, D. J., and B. T. Kao. 2002. “Integrated Sibling-Parent Group Intervention to Improve Sibling Knowledge and Adjustment to Chronic Illness and Disability.” *Journal of Pediatric Psychology* 27 (8): 711–716. 10.1093/jpepsy/27.8.711. 12403861

[CIT0028] Lummer-Aikey, S., and S. Goldstein. 2021. “Sibling Adjustment to Childhood Chronic Illness: An Integrative Review.” *Journal of Family Nursing* 27 (2): 136–153. 10.1177/1074840720977177. 33305651

[CIT0029] Marquis, S., M. V. Hayes, and K. McGrail. 2019. “Factors That May Affect the Health of Siblings of Children Who Have an Intellectual/Developmental Disability.” *Journal of Policy and Practice in Intellectual Disabilities* 16 (4): 273–286. 10.1111/jppi.12309.

[CIT0030] Martinez, B., P. Pechlivanoglou, D. Meng, B. Traubici, Q. Mahood, D. Korczak, M. Colasanto, S. Mahant, J. Orkin, and E. Cohen. 2022. “Clinical Health Outcomes of Siblings of Children with Chronic Conditions: A Systematic Review and Meta-Analysis.” *The Journal of Pediatrics* 250: 83.e8–92.e8. 10.1016/j.jpeds.2022.07.002. 35810772

[CIT0032] Meadan, H., J. B. Stoner, and M. E. Angell. 2010. “Review of Literature Related to the Social, Emotional, and Behavioral Adjustment of Siblings of Individuals with Autism Spectrum Disorder.” *Journal of Developmental and Physical Disabilities* 22 (1): 83–100. 10.1007/s10882-009-9171-7.

[CIT0033] Mellor, D. 2004. “Furthering the Use of the Strengths and Difficulties Questionnaire: Reliability with Younger Child Respondents.” *Psychological Assessment* 16 (4): 396–401. 10.1037/1040-3590.16.4.396. 15584800

[CIT0034] Moshagen, M., and M. Bader. 2024. “SemPower: General Power Analysis for Structural Equation Models.” *Behavior Research Methods* 56 (4): 2901–2922. 10.3758/s13428-023-02254-7. 37950114 PMC11522193

[CIT0035] Moss-Morris, R. 2013. “Adjusting to Chronic Illness: Time for a Unified Theory.” *British Journal of Health Psychology* 18 (4): 681–686. 10.1111/bjhp.12072. 24118260

[CIT0036] Moyson, T., and H. Roeyers. 2012. “The Overall Quality of My Life as a Sibling Is All Right, but of Course, It Could Always Be Better’. Quality of Life of Siblings of Children with Intellectual Disability: The Siblings’ Perspectives.” *Journal of Intellectual Disability Research* 56 (1): 87–101. 10.1111/j.1365-2788.2011.01393.x. 21366753

[CIT0037] Orm, S., T. Vatne, Y. B. Haukeland, W. K. Silverman, and K. Fjermestad. 2021. “The Validity of a Measure of Adjustment in Siblings of Children with Developmental and Physical Disabilities: A Brief Report.” *Developmental Neurorehabilitation* 24 (5): 355–358. 10.1080/17518423.2020.1869338. 33393399

[CIT0038] Ozolins, U., S. Hale, X. Cheng, A. Hyatt, and P. Schofield. 2020. “Translation and Back-Translation Methodology in Health Research – A Critique.” *Expert Review of Pharmacoeconomics & Outcomes Research* 20 (1): 69–77. 10.1080/14737167.2020.1734453. 32089017

[CIT0039] Park, C. G. 2023. “Implementing Alternative Estimation Methods to Test the Construct Validity of Likert-Scale Instruments.” *Korean Journal of Women Health Nursing* 29 (2): 85–90. 10.4069/kjwhn.2023.06.14.2. 37415477 PMC10326553

[CIT0040] Pinquart, M. 2023. “Behavior Problems, Self-Esteem, and Prosocial Behavior in Siblings of Children with Chronic Physical Health Conditions: An Updated Meta-Analysis.” *Journal of Pediatric Psychology* 48 (1): 77–90. 10.1093/jpepsy/jsac066. 35950954

[CIT0041] Pokropek, A., E. Davidov, and P. Schmidt. 2019. “A Monte Carlo Simulation Study to Assess The Appropriateness of Traditional and Newer Approaches to Test for Measurement Invariance.” *Structural Equation Modeling* 26 (5): 724–744. 10.1080/10705511.2018.1561293.

[CIT0042] Riley, A. W. 2004. “Evidence That School-Age Children Can Self-Report on Their Health.” *Ambulatory Pediatrics* 4 (4 Suppl): 371–376. 10.1367/A03-178R.1. 15264962

[CIT0043] Sahler, O. J., and P. J. Carpenter. 1989. “Evaluation of a Camp Program for Siblings of Children with Cancer.” *American Journal of Diseases of Children* 143 (6): 690–696. 10.1001/archpedi.1989.02150180068023. 2729214

[CIT0044] Schumann, A., T. M. Vatne, and K. W. Fjermestad. 2024. “What Challenges Do Siblings of Children with Chronic Disorders Express to Their Parents? A Thematic Analysis of 73 Sibling-Parent Dialogues.” *Journal of Pediatric Nursing* 76: 91–98. 10.1016/j.pedn.2024.01.032. 38367476

[CIT0045] Schweizer, K. 2010. “Some Guidelines Concerning the Modeling of Traits and Abilities in Test Construction.” *European Journal of Psychological Assessment* 26 (1): 1–2. 10.1027/1015-5759/a000001.

[CIT0046] Shi, D., T. Lee, A. J. Fairchild, and A. Maydeu-Olivares. 2020. “Fitting Ordinal Factor Analysis Models with Missing Data: A Comparison Between Pairwise Deletion and Multiple Imputation.” *Educational and Psychological Measurement* 80 (1): 41–66. 10.1177/0013164419845039. 31933492 PMC6943991

[CIT0047] Speyer, L. G., B. Auyeung, and A. L. Murray. 2023. “Longitudinal Invariance of the Strengths and Difficulties Questionnaire Across Ages 4 to 16 in the ALSPAC Sample.” *Assessment* 30 (6): 1884–1894. 10.1177/10731911221128948. 36254666 PMC10363935

[CIT0048] Stark, S., O. S. Chernyshenko, and F. Drasgow. 2006. “Detecting Differential Item Functioning with Confirmatory Factor Analysis and Item Response Theory: Toward a Unified Strategy.” *The Journal of Applied Psychology* 91 (6): 1292–1306. 10.1037/0021-9010.91.6.1292. 17100485

[CIT0049] Taylor, V., P. Fuggle, and T. Charman. 2001. “Well Sibling Psychological Adjustment to Chronic Physical Disorder in a Sibling: How Important Is Maternal Awareness of Their Illness Attitudes and Perceptions?”.” *Journal of Child Psychology and Psychiatry, and Allied Disciplines* 42 (7): 953–962. 10.1111/1469-7610.00791. 11693590

[CIT0050] Tsai, H.-W. J., K. Cebula, S. H. Liang, and S. Fletcher-Watson. 2018. “Siblings’ Experiences of Growing up with Children with Autism in Taiwan and the United Kingdom.” *Research in Developmental Disabilities* 83: 206–216. 10.1016/j.ridd.2018.09.001. 30248583

[CIT0051] Veerman, L. K. M., A. M. Willemen, S. D. M. Derks, A. A. J. Brouwer-van Dijken, and P. S. Sterkenburg. 2023. “The Effectiveness of the Serious Game ‘Broodles’ for Siblings of Children with Intellectual Disabilities and/or Visual Impairment: Study Protocol for a Randomized Controlled Trial.” *Trials* 24 (1): 336. 10.1186/s13063-023-07358-1. 37198687 PMC10190022

[CIT0052] Veerman, Linda K. M., Agnes M. Willemen, Stian Orm, Dr., Torun M. Vatne, Yngvild Haukeland, Paula Sterkenburg, and Krister Fjermestad. 2023. “Cross-country Psychometric Study of the Negative Adjustment Scale to Measure Siblings’ Adjustment to Their Brother’s or Sister’s Disability.” *OSF*. 10.17605/OSF.IO/5VNWB.

[CIT0053] Wofford, J. R., and R. G. Carlson. 2017. “A Literature Review and Case Study on the Strengths and Struggles of Typically Developing Siblings of Persons with Disabilities.” *The Family Journal* 25 (4): 398–406. 10.1177/1066480717732167.

[CIT0054] Wolff, B., I. Magiati, R. Roberts, E. Pellicano, and E. J. Glasson. 2022. “Risk and Resilience Factors Impacting the Mental Health and Wellbeing of Siblings of Individuals with Neurodevelopmental Conditions: A Mixed Methods Systematic Review.” *Clinical Psychology Review* 98: 102217. 10.1016/j.cpr.2022.102217. 36368218

[CIT0055] Wolff, B., I. Magiati, R. Roberts, R. Skoss, and E. J. Glasson. 2023. “Psychosocial Interventions and Support Groups for Siblings of Individuals with Neurodevelopmental Conditions: A Mixed Methods Systematic Review of Sibling Self-Reported Mental Health and Wellbeing Outcomes.” *Clinical Child and Family Psychology Review* 26 (1): 143–189. 10.1007/s10567-022-00413-4. 36175605 PMC9879846

[CIT0056] Wolff, B., V. R. Franco, I. Magiati, C. F. Pestell, and E. J. Glasson. 2023. “Neurocognitive and Self-Reported Psychosocial and Behavioral Functioning in Siblings of Individuals with Neurodevelopmental Conditions: A Study Using Remote Self-Administered Testing.” *Journal of Clinical and Experimental Neuropsychology* 45 (5): 513–536. 10.1080/13803395.2023.2259042. 37779193

