# Supplementary material for: Measuring adjustment of siblings of children with disabilities: psychometric properties across translations, age groups and informants
Source: Int J Dev Disabil. 2024 Oct 9;71(1):4–17. doi: 10.1080/20473869.2024.2411511 (PMC11774191; doi:10.1080/20473869.2024.2411511)
Supplement: Supplemental Material [file YJDD_A_2411511_SM4904.docx]

Table S1. Item endorsement frequencies and item-rest correlations of the Norwegian child report version per group (8-11/12-16 years).

| Item | Item-rest *r* | *Never* (1) | *A little* (2) | *Sometimes* (3) | *A lot* (4) | Missing |
| --- | --- | --- | --- | --- | --- | --- |
| 1.* | **.10/.30** | 41.8/30.7% | 18.4/21.8% | 29.6/29.7% | 10.2/17.8% | 0/0 |
| 2. | .60/.67 | 21.9/26.7% | 31.6/26.7% | 26.5/33.7% | 19.9/12.9% | 0/0 |
| 3. | **.39/.55** | 57.1/50.5% | 26.0/29.7/% | 15.3/14.9% | 1.5/5.0% | 0/0 |
| 4. | .51/.55 | 45.4/52.0% | 27.0./24% | 20.4/18% | 7.1/6.0% | 0/1 |
| 5. | .44/.52 | 15.4/11.9% | 41.8/42.6% | 29.1/29.7% | 13.3/15.8% | 1/0 |
| 6.* | **-.05/.16** | 6.1/0.0% | 14.3/80.0% | 25.0/20.0% | 54.6/72.0% | 0/1 |
| 7. | **.33/.63** | 40.8/38.6% | 26.0/23.8% | 24.0/20.8% | 9.2/16.8% | 0/0 |
| 8. | **.40/.54** | 20.9/26.3% | 23.0/29.3% | 31.1/13.1% | 25.0/31.3% | 0/2 |
| 9a. | **.16/.01** | 36.7/48.5% | 37.8/32.7% | 20.4/14.9% | 5.1/4.0% | 0/0 |
| 10. | .36/.35 | 8.2/9.9% | 19.9/18.8% | 28.6/33.7% | 43.4/37.6% | 0/0 |
| 11. | .30/.31 | 29.9/43.6% | 24.2/32.7% | 23.7/17.8% | 22.2/5.9% | 2/0 |
| 12. | .46/.46 | 18.0/25.7% | 32.3/30.7% | 28.2/28.7% | 21.5/14.9% | 1/0 |
| 13. | **.38/.25** | 77.0/83.2% | 9.2/8.9% | 8.2/6.9% | 5.6/1.0% | 0/0 |
| 14. | .31/.29 | 94.8/96.0% | 2.1/2.0% | 2.1/2.0% | 1.0/0.0% | 3/0 |
| 15. | .36/.43 | 80.5/73.3% | 13.9/17.8% | 5.6/8.9% | 0.0/0.0% | 1/0 |
| 16. | **.38/.22** | 53.3/55.5% | 28.7/22.8% | 12.3/18.8% | 5.6/3.0% | 1/0 |
| 17.* | .15/.20 | 3.6/3.0% | 20.5/18.2% | 35.4/35.4% | 40.5/43.4% | 1/2 |
| 18. | .35/.43 | 16.4/17.0% | 24.1/23.0% | 30.8/28.0% | 28.7/32.0% | 1/1 |

*Note.* Valid percentages reported. Differences in item-rest correlations of ≥ 0.10 are indicated with bold face. *Non-reversed item frequencies reported. Reversed items are used for the item-rest correlations.

Table S2. Inter-item correlations of the Norwegian child report version (N = 297).

|  | 1. |  | 2. |  | 3. |  | 4. |  | 5. |  | 6. |  | 7. |  | 8. |  | 9a. |  | 10. |  | 11. |  | 12. |  | 13. |  | 14. |  | 15. |  | 16. |  | 17. |  | 18. |  |
| --- | --- | --- | --- | --- | --- | --- | --- | --- | --- | --- | --- | --- | --- | --- | --- | --- | --- | --- | --- | --- | --- | --- | --- | --- | --- | --- | --- | --- | --- | --- | --- | --- | --- | --- | --- | --- |
| 1. | — |  |  |  |  |  |  |  |  |  |  |  |  |  |  |  |  |  |  |  |  |  |  |  |  |  |  |  |  |  |  |  |  |  |  |  |
| 2. | -.13 | * | — |  |  |  |  |  |  |  |  |  |  |  |  |  |  |  |  |  |  |  |  |  |  |  |  |  |  |  |  |  |  |  |  |  |
| 3. | -.10 |  | .33 | ** | — |  |  |  |  |  |  |  |  |  |  |  |  |  |  |  |  |  |  |  |  |  |  |  |  |  |  |  |  |  |  |  |
| 4. | -.07 |  | .51 | ** | .31 | ** | — |  |  |  |  |  |  |  |  |  |  |  |  |  |  |  |  |  |  |  |  |  |  |  |  |  |  |  |  |  |
| 5. | -.13 | * | .50 | ** | .28 | ** | .38 | ** | — |  |  |  |  |  |  |  |  |  |  |  |  |  |  |  |  |  |  |  |  |  |  |  |  |  |  |  |
| 6. | .06 |  | .01 |  | .05 |  | .04 |  | .00 |  | — |  |  |  |  |  |  |  |  |  |  |  |  |  |  |  |  |  |  |  |  |  |  |  |  |  |
| 7. | -.09 |  | .35 | ** | .32 | ** | .28 | ** | .23 | ** | .03 |  | — |  |  |  |  |  |  |  |  |  |  |  |  |  |  |  |  |  |  |  |  |  |  |  |
| 8. | -.13 | * | .36 | ** | .27 | ** | .29 | ** | .20 | ** | -.03 |  | .31 | ** | — |  |  |  |  |  |  |  |  |  |  |  |  |  |  |  |  |  |  |  |  |  |
| 9a. | .02 |  | .18 | * | .11 |  | .17 | * | .22 | ** | .04 |  | .16 | * | .07 |  | — |  |  |  |  |  |  |  |  |  |  |  |  |  |  |  |  |  |  |  |
| 10. | -.15 | * | .42 | ** | .14 | * | .21 | ** | .33 | ** | .18 | * | .24 | ** | .25 | ** | .01 |  | — |  |  |  |  |  |  |  |  |  |  |  |  |  |  |  |  |  |
| 11. | -.13 | * | .29 | ** | .05 |  | .27 | ** | .18 | * | -.09 |  | .03 |  | .18 | * | .02 |  | .27 | ** | — |  |  |  |  |  |  |  |  |  |  |  |  |  |  |  |
| 12. | -.04 |  | .26 | ** | .26 | ** | .25 | ** | .13 | * | -.12 | * | .26 | ** | .28 | ** | .02 |  | .20 | ** | .26 | ** | — |  |  |  |  |  |  |  |  |  |  |  |  |  |
| 13. | .01 |  | .29 | ** | .11 | * | .37 | ** | .21 | ** | -.00 |  | .09 |  | .23 | ** | .07 |  | .11 |  | .27 | ** | .21 | ** | — |  |  |  |  |  |  |  |  |  |  |  |
| 14. | -.05 |  | .19 | * | .22 | ** | .14 | * | .18 | * | -.03 |  | .19 | ** | .14 | * | .07 |  | .15 | * | .19 | * | .17 | * | .21 | ** | — |  |  |  |  |  |  |  |  |  |
| 15. | .07 |  | .29 | ** | .32 | ** | .20 | ** | .17 | * | -.05 |  | .21 | ** | .21 | ** | .04 |  | .16 | * | .01 |  | .34 | ** | .19 | ** | .07 |  | — |  |  |  |  |  |  |  |
| 16. | -.08 |  | .23 | ** | .15 | * | .17 | * | .19 | * | -.09 |  | .11 |  | .16 | * | .00 |  | .08 |  | .08 |  | .30 | ** | .12 | * | .10 |  | .31 | ** | — |  |  |  |  |  |
| 17. | .21 | ** | -.06 |  | -.20 | ** | -.09 |  | -.02 |  | .08 |  | -.12 | * | -.12 | * | .12 | * | .00 |  | .01 |  | -.09 |  | -.05 |  | -.08 |  | -.11 |  | -.14 | * | — |  |  |  |
| 18. | -.05 |  | .26 | ** | .26 | ** | .26 | ** | .14 | * | .01 |  | .30 | ** | .19 | ** | -.02 |  | .17 | * | .08 |  | .36 | ** | .13 | * | .18 | * | .24 | ** | .24 | ** | -.09 |  | — |  |

*Note.* Non-reversed items are used. * *p* < 0.05, ** p < 0.001.

Table S3. Inter-item correlations of the Dutch child and parent report version (N = 103/102).

|  | 1. | | 2. | | 3. | | 4. |  | 5. |  | 6. |  | 7. |  | 8. |  | 9a. |  | 9b. |  | 10. |  | 11. |  | 12. |  | 13. |  | 14. |  | 15. |  | 16. |  | 17. |  | 18. |  |
| --- | --- | --- | --- | --- | --- | --- | --- | --- | --- | --- | --- | --- | --- | --- | --- | --- | --- | --- | --- | --- | --- | --- | --- | --- | --- | --- | --- | --- | --- | --- | --- | --- | --- | --- | --- | --- | --- | --- |
| 1. | — |  |  |  |  |  |  |  |  |  |  |  |  |  |  |  |  |  |  |  |  |  |  |  |  |  |  |  |  |  |  |  |  |  |  |  |  |  |
| 2. | **-.11/-.25** | * | — |  |  |  |  |  |  |  |  |  |  |  |  |  |  |  |  |  |  |  |  |  |  |  |  |  |  |  |  |  |  |  |  |  |  |  |
| 3. | .18**/-.11 |  | **.21*/.46** | ****** | — |  |  |  |  |  |  |  |  |  |  |  |  |  |  |  |  |  |  |  |  |  |  |  |  |  |  |  |  |  |  |  |  |  |
| 4. | -.12/-.16 |  | .35**/.34 | ** | .37**/.29 | * | — |  |  |  |  |  |  |  |  |  |  |  |  |  |  |  |  |  |  |  |  |  |  |  |  |  |  |  |  |  |  |  |
| 5. | **-.06*/-.29** | ***** | **.26*/.50** | ****** | **.22*/.32** | ***** | .31*/.29 | * | — |  |  |  |  |  |  |  |  |  |  |  |  |  |  |  |  |  |  |  |  |  |  |  |  |  |  |  |  |  |
| 6. | **-.08/-.17** |  | .02/.01 |  | *-.08/.15* |  | -.04/-.01 |  | **.18/.08** |  | — |  |  |  |  |  |  |  |  |  |  |  |  |  |  |  |  |  |  |  |  |  |  |  |  |  |  |  |
| 7. | **-.05/-.17** |  | **.26*/.46** | ****** | **.20*/.31** | ***** | .30*/.37 | ** | **.16/.34** | ****** | -.04/-.07 |  | — |  |  |  |  |  |  |  |  |  |  |  |  |  |  |  |  |  |  |  |  |  |  |  |  |  |
| 8. | **-.18/-.09** |  | **.22*/.38** | ****** | .19/.20 | * | .10/.16 |  | .26*/.20 | * | *-.01/.05* |  | .22*/.21 | * | — |  |  |  |  |  |  |  |  |  |  |  |  |  |  |  |  |  |  |  |  |  |  |  |
| 9a. | -.21*/-.15 |  | *-.09/.06* |  | .14/.13 |  | *.08/-.05* |  | .26*/.19 | * | *-.01/.02* |  | .03/.19 |  | **.19/.05** |  | — |  |  |  |  |  |  |  |  |  |  |  |  |  |  |  |  |  |  |  |  |  |
| 9b. | -.04/-.02 |  | .24*/.26 | * | .24*/.18 |  | .17/.18 |  | .29*/.23 | * | **-.02/-.17** |  | **.13/.51** | ****** | .18/.17 |  | **.03/.15** |  | — |  |  |  |  |  |  |  |  |  |  |  |  |  |  |  |  |  |  |  |
| 10. | **.06/-.21** | ***** | **.29*/.51** | ****** | .19/.25 | * | **.09/.35** | ****** | **.22*/.44** | ****** | **-.01/.15** |  | .28*/.30 | * | **.22*.50** | ****** | **.18/.02** |  | **.28*/.16** |  | — |  |  |  |  |  |  |  |  |  |  |  |  |  |  |  |  |  |
| 11. | -.05/-.13 |  | **.18/.43** | ****** | **.10/.24** | ***** | .09/.06 |  | **.24*/.36** | ****** | -.08/-.04 |  | **.05/.14** |  | **.39**/.29** | ***** | -.08/-.05 |  | **.29*/.09** |  | .32**/.39 | ** | — |  |  |  |  |  |  |  |  |  |  |  |  |  |  |  |
| 12. | ***-.01/.15*** |  | .23*/.21 | * | **.36**/.17** |  | **.24*/.07** |  | **.21*/.10** |  | **-.10/-.26** | ***** | .22*/27 | * | **.09/.22** | ***** | *-.05/.05* |  | .52**/.53 | ** | .11/.12 |  | .09/.15 |  | — |  |  |  |  |  |  |  |  |  |  |  |  |  |
| 13. | *.06/-.05* |  | .44**/.21 | * | *.12/-.05* |  | .27*/.34 | ** | .13/.08 |  | **-.23*/-.33** | ****** | **.14/.29** | ***** | .03/.00 |  | *-.09/.04* |  | .18/.17 |  | *.03/-.04* |  | .09/.08 |  | .21*/.13 |  | — |  |  |  |  |  |  |  |  |  |  |  |
| 14. | -.04/-.06 |  | .07/.06 |  | .10/.07 |  | **.14/04** |  | **.16/.06** |  | **-.29*/-.13** |  | *-.04/.11* |  | **.19/.06** |  | **.28*/.03** |  | .17/.12 |  | .12/.12 |  | .10/.14 |  | .18/.11 |  | **.34**/.24** | ***** | — |  |  |  |  |  |  |  |  |  |
| 15. | *-.08/.07* |  | **.19/.33** | ****** | .19/.24 | * | .10/.05 |  | .12/.15 |  | **-.13/-.30** | ***** | *.18/.36* | **** | **.04/.29** | ***** | **.05/.21** | ***** | **.21*/.44** | ****** | **.08/.27** | ***** | .15/.16 |  | **.46**/.64** | ****** | .24*/.17 |  | .17/.13 |  | — |  |  |  |  |  |  |  |
| 16. | .03/.06 |  | .10/.18 |  | **.21*/.10** |  | **.20/.01** |  | .28*/.24 | * | -.17/-.20 | * | .10/.13 |  | .12/.20 | * | .04/.03 |  | .24*/.22 | * | .11/.14 |  | .23*/.16 |  | .27*/.32 | * | .24*/.19 |  | **.10/.21** | ***** | **.21*/.34** | ****** | — |  |  |  |  |  |
| 17. | -.06/-.10 |  | ***.37**/-.02*** |  | *.13/-.02* |  | **.29*/.03** |  | .24*/.19 |  | *-.03/.09* |  | .21*/.16 |  | .19/.11 |  | .14/.05 |  | .09/.06 |  | .06/.09 |  | .02/.09 |  | .14/.09 |  | ***.34**/-.07*** |  | ***.33**/-.06*** |  | *-.19/.15* |  | **.13/.02** |  | — |  |  |  |
| 18. | **-.04/-.36** | ****** | **.10/.45** | ****** | .20*/.29 | * | **.22*/.31** | ***** | .32*/.32 | * | **.22*/.08** |  | **.03/.33** | ****** | ***-.07/.17*** |  | .08/.02 |  | .17/.24 | * | .17/.22 | * | **.09/.20** | ***** | .27*/.19 |  | *-.06/.14* |  | *-.05/.09* |  | **.05/.16** |  | **.14/.03** |  | **.24*/.04** |  | — |  |

*Note.* Reported as child/parent version. Non-reversed items are used. Differences of ≥.1 are indicated with bold face. Differences in direction are indicated with italic face. * *p* < 0.05, ** p < 0.001.

Table S4. Inter-item correlations of the Norwegian child report version per group (8-11 years/12-16 years).

|  | 1. | | 2. | | 3. | | 4. |  | 5. |  | 6. |  | 7. |  | 8. |  | 9a. |  | 10. |  | 11. |  | 12. |  | 13. |  | 14. |  | 15. |  | 16. |  | 17. |  | 18. |  |  |  |
| --- | --- | --- | --- | --- | --- | --- | --- | --- | --- | --- | --- | --- | --- | --- | --- | --- | --- | --- | --- | --- | --- | --- | --- | --- | --- | --- | --- | --- | --- | --- | --- | --- | --- | --- | --- | --- | --- | --- |
| 1. | — |  |  |  |  |  |  |  |  |  |  |  |  |  |  |  |  |  |  |  |  |  |  |  |  |  |  |  |  |  |  |  |  |  |  |  |  |  |
| 2. | **-.06/-.26** | ***** | — |  |  |  |  |  |  |  |  |  |  |  |  |  |  |  |  |  |  |  |  |  |  |  |  |  |  |  |  |  |  |  |  |  |  |  |
| 3. | **-.07/-.17** |  | **.29**/.42** | ****** | — |  |  |  |  |  |  |  |  |  |  |  |  |  |  |  |  |  |  |  |  |  |  |  |  |  |  |  |  |  |  |  |  |  |
| 4. | -.07/-.06 |  | .53**/.48 | ** | **.25**/.42** | ****** | — |  |  |  |  |  |  |  |  |  |  |  |  |  |  |  |  |  |  |  |  |  |  |  |  |  |  |  |  |  |  |  |
| 5. | **-.08/.25** | ***** | **.00/.57** | ****** | .31**/.30 | * | **.34**/.44** | ****** | — |  |  |  |  |  |  |  |  |  |  |  |  |  |  |  |  |  |  |  |  |  |  |  |  |  |  |  |  |  |
| 6. | .04/.02 |  | *.04/-.11* |  | *.07/-.05* |  | .*13/-.15* |  | **.26**/.01** |  | — |  |  |  |  |  |  |  |  |  |  |  |  |  |  |  |  |  |  |  |  |  |  |  |  |  |  |  |
| 7. | ***.03.-.32*** | ******* | **.31**/.42** | ****** | **.24**/.44** | ****** | **.19*/.46** | ****** | **.09/.47** | ****** | ***.07/-.14*** |  | — |  |  |  |  |  |  |  |  |  |  |  |  |  |  |  |  |  |  |  |  |  |  |  |  |  |
| 8. | **-.07/-.24** | ***** | .34**/.38 | ** | .25**/.30 | * | **.24**/.38** | ****** | **.17*/.28** | ***** | ***.04/-.17*** |  | **.21*/.49** | ****** | — |  |  |  |  |  |  |  |  |  |  |  |  |  |  |  |  |  |  |  |  |  |  |  |
| 9a. | **-.04/.17** |  | .16*/.19 |  | **.18*/.01** |  | **.20*/.09** |  | .22**/.23 | * | .07/.07 |  | **.24**/.02** |  | *.12/-.04* |  | — |  |  |  |  |  |  |  |  |  |  |  |  |  |  |  |  |  |  |  |  |  |
| 10. | -.12/-.19 |  | .43**/.41 | ** | **.07/.27** | ***** | **.26**/.09** |  | .33**/.35 | ** | .20*/.18 |  | .22*/.28 | * | .22*/.30 | * | *-.04/.09* |  | — |  |  |  |  |  |  |  |  |  |  |  |  |  |  |  |  |  |  |  |
| 11. | -.13/-.07 |  | .27**/.31 | * | .06/.07 |  | **.21*/.38** | ****** | .22**/.14 |  | **-.01/-.17** |  | **.00/.15** |  | .15*/.23 | * | *-.02/.04* |  | **.30**/.18** |  | — |  |  |  |  |  |  |  |  |  |  |  |  |  |  |  |  |  |
| 12. | ***.01/-.11*** |  | .24**/.19 |  | **.23*/.35** | ****** | **.27**/.19** |  | **.10/.20** | ***** | -.09/-.16 |  | **.21*/.38** | ****** | .25**/.33 | ** | *.03/-.04* |  | .22*/.17 |  | .26**/.21 | * | — |  |  |  |  |  |  |  |  |  |  |  |  |  |  |  |
| 13. | -.00/.07 |  | .28**/.37 | ** | .10/.19 |  | .35**/.42 | ** | .23**/.18 |  | .04/-.07 |  | **.06/.20** | ***** | .21**/.26 | * | *.12/-.10* |  | **.13/.04** |  | .27**/.20 | * | .***29**/-.02*** |  | — |  |  |  |  |  |  |  |  |  |  |  |  |  |
| 14. | **-.01 /-.15** |  | .18*/.17 |  | **.18*/.35** | ****** | .15*/.11 |  | .20**/.14 |  | -.00/-.09 |  | **.24**/.09** |  | .14*/.14 |  | *.10/-.02* |  | .16*/.13 |  | .20*/.13 |  | .19*/.13 |  | .20*/.23 | * | — |  |  |  |  |  |  |  |  |  |  |  |
| 15. | .19*/-.15 |  | **.25**/.37** | ****** | .28**/.37 | ** | .24**/.16 |  | .14*/.21 | * | **-.04/-.18** |  | **.17*/.27** | ****** | .22*/.21 | * | .06/.02 |  | .14/.20 |  | .01/.07 |  | .33**/.39 | ** | **.25**/.12** |  | .10/.04 |  | — |  |  |  |  |  |  |  |  |  |
| 16. | **.00/-.23** | ***** | .26**/.17 |  | **0.23*/.03** |  | .16*/.18 |  | **.23**/.11** |  | -.11/-.04 |  | .11/.11 |  | **.21*/.07** |  | *-.03/.08* |  | ***.15*/-.05*** |  | .07/.10 |  | .31**/.27 | * | **.15*/.05** |  | **.13/.03** |  | .33**/.30 | * | — |  |  |  |  |  |  |  |
| 17. | .18*/.25 | *** | .*05/-.09* |  | **-.12/-.35** | ****** | -.08/-.09 |  | *-.04/.02* |  | .08/.07 |  | **-.09/-.19** |  | -.10/-.14 |  | **.07/.22** | ***** | *.03/-.04* |  | *.06/-.09* |  | -.06/-.14 |  | -.05/-.06 |  | **-.02/-.24** |  | **-.08/-.18** |  | ***-.24**/.07*** |  | — |  |  |  |  |  |
| 18. | **.00/-.14** |  | **.22*/.32** | ******* | **.20*/.37** | ******** | .26**/.25 | *** | **.09/.23** | ******* | ***.05/-.16*** |  | .29**/.32 | *** | **.13/.29** | ******* | -.00/-.05 |  | .15*/.21 | * | .10/.07 |  | .34**/.40 | ** | .15*/.07 |  | .17*/.22 |  | .26**/.20 | *** | **.27**/.17** |  | -.09/-.09 |  | — |  |  |  |

*Note.* Non-reversed items are used. Differences of ≥.1 are indicated with bold face. Differences in direction are indicated with italic face. * *p* < 0.05, ** p < 0.001.

Table S5. Factor loadings and residual variances of the parent and child report versions, including all items.

|  | Standardized Factor Loading | | | | Residual Variance | |
| --- | --- | --- | --- | --- | --- | --- |
| Item | Parents | 95% *C.I.* | Children | 95% *C.I.* | Parents | Children |
| NAS1* | .262 | .168-.356 | .128 | .003-.252 | .931 | .984 |
| NAS2 | .805 | .717-.894 | .608 | .502-.715 | .351 | .630 |
| NAS3 | .496 | .410-.582 | .531 | .429-.633 | .754 | .718 |
| NAS4 | .466 | .369-.564 | .635 | .521-.749 | .783 | .597 |
| NAS5 | .650 | .570-.730 | .553 | .448-.658 | .577 | .694 |
| NAS6* | .108 | .008-.207 | .091 | -.017-.200 | .988 | .992 |
| NAS7 | .659 | .576-.742 | .486 | .370-.602 | .565 | .764 |
| NAS8 | .501 | .407-.594 | .411 | .307-.516 | .749 | .831 |
| NAS9b | .659 | .576-.742 | .653 | .551-.756 | .566 | .573 |
| NAS10 | .662 | .579-.745 | .418 | .308-.529 | .561 | .825 |
| NAS11 | .478 | .386-.570 | .387 | .282-.492 | .771 | .850 |
| NAS12 | .643 | .559-.727 | .691 | .590.-792 | .586 | .523 |
| NAS13 | .458 | .320-.595 | .577 | .437-.717 | .791 | .667 |
| NAS14 | .379 | .182-.575 | .414 | .282-.545 | .857 | .829 |
| NAS15 | .651 | .564-.738 | .458 | .351-.566 | .576 | .790 |
| NAS16 | .370 | .276-.465 | .424 | .324-.524 | .863 | .820 |
| NAS17 | .145 | .048-.241 | .543 | .437-.649 | .979 | .705 |
| NAS18 | .534 | .430-.638 | .319 | .220-.419 | .715 | .898 |

*Note.* Standardized factor loadings are reported. *Reversed item
